# Access to Oral health care: a focus on dental caries treatment provision in Enugu Nigeria

**DOI:** 10.1186/s12903-020-01135-1

**Published:** 2020-05-19

**Authors:** Nkolika Uguru, Obinna Onwujekwe, Udochukwu Ugochukwu Ogu, Chibuzo Uguru

**Affiliations:** 1 Department of Preventive Dentistry, Faculty of Dentistry, College of Medicine, University of Nigeria, Enugu Campus, Enugu, Nigeria; 2 Department of Health Administration and Management, Faculty of Health Sciences and Technology, University of Nigeria, Enugu Campus, Enugu, Nigeria; 3 Health Policy Research Group, Department of Pharmacology and Therapeutics, College of Medicine, University of Nigeria, Enugu Campus, Enugu, Nigeria; 4 Department of Oral and Maxillofacial Surgery, Faculty of Dentistry, College of Medicine, University of Nigeria, Enugu Campus, Enugu, Nigeria

**Keywords:** Dental caries, Oral health, Caries treatment/services, Oral healthcare provision

## Abstract

**Background:**

Dental caries, despite improvement in oral health across the globe, is still a large contributor to the global burden of oral diseases and a major public health concern. In Enugu state, Nigeria, there is minimal access to adequate and proper oral health care. This study examined the determinants of dental caries treatment provision and the challenges of providing equitable access to oral health care.

**Method:**

This was a mixed-method cross-sectional descriptive urban-rural study conducted in selected oral health facilities offering primary oral health care in Enugu state. The study was conducted in two phases over a 2 month period. Quantitative data was initially collected from all selected oral health care providers using a survey questionnaire format after which qualitative data were collected through in-depth interviews of heads of the selected oral health facilities. The determinants of dental caries treatment services were explored with a focus on provider behavior, cost of dental services, human resource availability and availability of dental equipment.

**Results:**

Quantitative findings show that to a larger extent, the cost of raw materials (100%), human resources (98.1%), infection control resources (98.1%), geographical location (98.1), Government policies (88%) and the price of other goods (80.8%) influence provision of dental caries treatment services. Qualitative results show that location and number of oral health facilities, government funding and policies for oral health, cost of dental equipment and materials, the ability of consumers to pay, human resource availability and consumer awareness of oral health are also factors that influence the provision of dental caries treatment services.

**Conclusion:**

Adequate access to oral health care services is a major concern that affects all aspects of healthcare and a determining factor in the country’s drive to achieve universal health coverage. In order to address this, oral health facilities need to be strategically located and have adequate materials, equipment and skilled staff. There is a need to incorporate oral health into the general health care system and improve government policies and funding for oral health.

## Background

Access to oral health care in Nigeria is poor [1] and efforts made to improve access to oral health care in Nigeria have been largely unsuccessful [2]. Poor integration of oral health into general health has been the bane of the Nigerian health system despite the seeming progress in oral health evidenced by the introduction of the 2012 Oral health policy [2]. A few studies found that the system falls short of many desirable attributes. These studies show that the health system, is neither efficient nor effective thus available resources in many areas, are overstretched and grossly inadequate [1, 3]. However, in a bid to improve equitable access to oral health care services, The Nigerian government has included oral health care in the National health insurance scheme [3].

Access to oral health care can be referred to as the ability of a patient to use oral health care [4]. There are many factors that have been known to influence access to oral health care in Nigeria and these can be categorized into contextual and individual factors. The contextual factors include the inability to obtain dental insurance, shortage and mal-distribution of dentists, the cost associated with dental care, insufficient professional input on evidence-based guidelines, Rurality, lack of interdisciplinary collaborations and a complex oral health system that can be difficult to navigate [4–6]. The individual factors are anxiety and fear associated with dental care, low oral health literacy, perceptions and misconceptions that exist about oral health care [4, 5].

Olusile [7], pointed out that to reduce the cost of dental treatment, health insurance should be made available. He further pointed out the role of dentists in the dissemination of oral health education, rural community practice, use of allied or non-dental personnel, volunteers and research. The current study will attempt to identify more contextual factors and also, find out how most of these factors determine treatment service provision and its overall influence on access.

In many developing countries, the shortage and unequal distribution of dentists mean that carious teeth will remain untreated [8]. In the majority of Africa, there is little or no access to adequate and proper oral health care [9]. The ratio of dentists to a population (in Africa) is quite low at 1:150,000 when compared with high-income countries with a dentist population ratio of 1:2000 [9]. A study in 2012, in Nigeria, showed that there are about 4125 registered dentists, which is about 40,000 people to 1 dentist [9]. However, by 2017, the population of Nigeria was estimated at 193 million and the dentist population ratio was reported to have dropped to 1 dentist for every 38,600 people (1400 less of the 2012 figure) [10].

An oral health referendum held by the International Dental Federation, International Association of Dental Research and the World Health Organization revisited the global oral health goals set in the year 2000 [5]. The focus of the referendum was on improving access to oral health care so as to reduce morbidity and mortality from oral diseases, promote evidence-based oral health policies and reduce disparities in access to oral healthcare [11]. However, this has not really been actualized in Nigeria [1]. The reason for this being that the little resources assigned to the health sector are mainly directed towards life-threatening conditions such as HIV/AIDS, tuberculosis, and malaria rather than dental caries and other oral diseases [12, 13]. In addition, the strategies to improve access documented in the country’s oral health policy are yet to be fully implemented [1, 13, 14].

Health care resources are scarce, and in the allocation of these scarce resources, decision-makers in Nigeria, pay little or no attention to oral health prevention programs or dental treatment programs [13]. Thus, in Nigeria, oral health care is seen as insignificant when compared to other areas of health and proper attention has not been given to oral health issues [2]. The country lacks a coordinated system of collecting health data, especially in oral health, thus, making an accurate assessment of the oral health care system difficult [2].

Dental caries, despite improvement in oral health across the globe, still contributes to the global burden of disease and is a major public health concern [1]. However, there has been a decrease in the occurrence of dental caries in some developed countries [8]. The decline has been attributed to both improved oral hygiene practices and the use of fluoride in oral health care [8]. Although evidence shows that in African countries like Nigeria, caries prevalence is low [15–17], there are limited national studies on caries incidence only fragmented studies have been carried out in specific states [8]. The most recent national estimate of caries prevalence shows that prevalence in Nigeria is between 4 and 40% in adults with a higher 40% being found in the urban area. This wide range is attributed to socioeconomic differences between rural and urban dwellers with urban dwellers being able to afford a more caries prone westernized diet [2, 13]. There is limited research on dental caries treatment services in Enugu, Nigeria. There is also limited literature on the challenges of providing oral health care. This study will seek to add to knowledge by examining the determinants of dental caries treatment service provision in Enugu State.

## Methods

This study was conducted in Enugu State Nigeria. Enugu state is one of the 36 states of the Federal Republic of Nigeria and is divided into 17 Local Government Areas (LGAs). Four urban and thirteen rural LGAs [14]. The study was conducted in three local government areas in Enugu State. (Enugu East which is urban with a projected population of 374,100 and Nsukka and Awgu LGAs which are both rural LGAs with a projected population of 417,700 and 390,681 respectively) [18].

The state operates a mixed public and private system of health care. Public oral health care can be accessed at three levels namely primary, secondary and tertiary. At the primary care level, Public oral health facilities offer only primary oral health care which is mostly prevention and basic services such as scaling and polishing, simple extractions, simple teeth restorations, oral health education and promotion services. The secondary level consists of oral health facilities, with or without laboratory services. Secondary level care includes treatment of more advanced cases of oral diseases and offers more advanced treatment of dental care. The cadre of staff employed is mostly general dental practitioners, dental officers, dental therapists, dental nurses and technologists. The third level represents the highest level of oral health care and these include the teaching hospitals and specialist hospitals. They offer more specialized and advanced treatment of oral diseases. For the private clinics, they mostly offer both primary and secondary level care with or without laboratories attached.

This was a mixed-method cross-sectional descriptive urban-rural study conducted in oral health facilities offering primary oral health care in Enugu State. The study comprised a survey of public oral health facilities and private dental clinics offering primary oral health care in the state. The study, which was carried out over 2 months, from November 2018 to January 2019 was conducted in two phases starting with the quantitative followed by the qualitative study.

In the first phase, a multi-stage sampling technique was used starting with the selection of the LGA’s followed by the selection of the oral health care facilities and finally the oral health care service providers. Nsukka LGA was purposively selected as the only LGA in the state with a public health facility offering primary oral healthcare services, Awgu LGA was purposively selected because it accommodates the principal referral hospital for oral healthcare in the state and Enugu East LGA was randomly selected from a list of urban LGAs in the state.

The next stage was the selection of oral health care facilities. In the rural LGA, Obukpa health center was purposively selected as the only rural public health facility in the state delivering primary oral health care. In the selected urban LGA, Federal school of dental technology was purposively selected because it is the only public health facility offering oral healthcare services. Two private dental clinics were randomly selected from both the rural and urban LGA’s respectively while the principal referral center for the two facilities - University of Nigeria Teaching Hospital, was purposively selected.

At the final stage, all frontline oral service providers in all the selected oral health facilities were surveyed. There are 65 oral health care providers in Enugu State [19] and the total number of oral health care providers in the selected facilities was 52. This was done to enable an adequate number of participants to take part in the study because of the limited number of dental professionals in the state. Thus the sample size for dental providers who participated in the quantitative study was 52. A close-ended interviewer-administered questionnaire was used until the stipulated sample size was attained.

In the second phase, a purposive sampling method was used to collect qualitative information from all heads of oral health facilities or their representatives through in-depth interviews using a structured interview guide. There were 7 respondents in total and saturation was achieved. The in-depth interview of key informants was used to investigate the perception of dental care professionals on dental caries treatment service provision. It specifically looked at the type of service provided, challenges in service provision, the influence of government policies and taxes on the provision of treatment services, as well as the influence of different health financing mechanisms.

### Data analysis

For the quantitative study, data were analyzed using SPSS version 20. A descriptive analysis was initially conducted to provide socio-demographic information of respondents. A bivariate analysis (chi-square test) was done to test the association across two population groups (public and private oral health facilities). A *p*-value of 0.05 was accepted as statistically significant.

In order to test the mean the cost of different dental procedures across two groups (public and private). The mean cost of each procedure in the two groups was compared using independent samples t-test (Levene’s test) to find out the existence of variations in means between the public and private facilities. Two hypotheses were used in the testing the means; (**H**_**0**_): there is no significant difference in the cost of providing dental caries treatment service in the public and private dental facilities and (**H**_**1**_): there is a significant difference in the cost of providing dental caries treatment service in the public and private dental facilities. This entails that if the *p*-value is less than our chosen significance level, we can reject the null hypothesis and accept our alternative hypothesis.

**Fig. 1:**
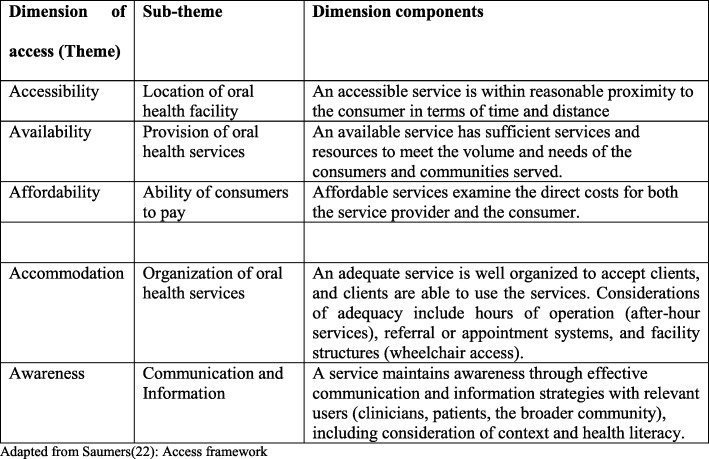
Framework for analyzing of dental caries treatment services provision. Adapted from Saumers [22]: Access framework

For the qualitative study, an in-depth interview guide was developed, pretested and revised before use in the study. The interviews were conducted face to face in the dental facilities using audio recorders and later transcribed verbatim. Transcriptions were analyzed using thematic content analysis. Themes were derived based on the access framework adapted from three different authors, namely Penchasky and Thomas [20], Levesque and Russell [21] and Saumers [22] as shown in Fig. 1. Relevant themes were derived with a focus on supply based on each dimension of access, the framework was reviewed and revised, quotations and translations were checked and then authors developed an explanatory narrative for this paper.

## Results

Table 1 below shows that the majority of the respondents are from public hospitals and they are mainly dentists and dental therapists. Respondents in the public hospitals comprise both tertiary and primary health center staff. The primary health center in the rural area has just one dentist. While the tertiary facility has 35 respondents.

**Table 1:** Provider characteristics

Variables	Total f (%)	Public facility f (%)	Private facility f (%)	X^**2**^ (***P***-value)
**Geographical location**				12.745 (0.001)
Urban	15 (28.8)	9 (17.3)	6 (11.5)	
Rural	37 (71.2)	36 (67.3)	1 (1.9)	
Total	52 (100.0)	45 (86.5)	7 (13.5)	
**Facility type**				16.654 (0.000)
Tertiary Hospital	35 (67.3)	35 (67.3)	0 (0.0)	
*Rural Health Centre	1 (1.9)	1 (1.9)	–	
Dental clinic	16 (30.8)	9 (17.3)	7 (13.5)	
Total	52 (100.0)	45 (86.5)	7 (13.5)	
**Cadre of Respondents**				1.378 (0.711)
Doctor	1 (1.9)	1 (1.9)	0 (0.0)	
Dentist	31 (59.6)	28 (53.8)	3 (5.8)	
Dental nurse	4 (7.7)	3 (5.8)	1 (1.9)	
Dental Therapist	16 (30.8)	13 (25.0)	3 (5.8)	
Total	52 (100.0)	45 (86.5)	7 (13.5)	
**Highest education level**				2.245 (0.523)
Diploma	20 (38.5)	16 (30.8)	4 (7.7)	
Bachelor of Dental Surg	26 (50.0)	24 (46.2)	2 (3.8)	
Master’s degree	4 (7.7)	3 (5.8)	1 (1.9)	
Post-grad. Fellowship	2 (3.8)	2 (3.8)	0 (0.0)	
Total	52 (100.0)	45 (86.5)	7 (13.5)	

*Public oral facility in the rural area comprises respondents in both tertiary and primary health facilities

Table 2, shows there is no significant difference in facility opening days and times across both facility types (*p* < 0.05). Only the tertiary hospitals are open 24 h. However, private facilities are open for much longer. The average patient load is more in the public health facility than private with the highest service being tooth extraction (*P* > 0.05). The majority of the respondents across both the public and private dental facilities indicate the existence of a referral mechanism and the patients are usually referred to the tertiary health facility.

**Table 2:** Availability of dental services and provision of treatment

Variables	Total n (%) *N* = 52	Public facility n (%)	Private facility n (%)	X^2^ (P-Value)
Facility opening days				44.48 (0.000)
Monday to Friday	15 (28.8)	15 (28.8)	0 (0.0)	
Monday to Saturday	8 (15.8)	1 (1.9)	7 (13.5)	
Monday to Sunday (Tertiary)	28 (55.8)	29 (55.8)	0 (0.0)	
Facility opening times				38.26 (0.000)
24 h (Tertiary)	34 (65.4)	34 (65.4)	0 (0.0)	
8 am-4 pm	10 (19.2)	8 (15.4)	2 (3.8)	
8 am-6 pm	2 (3.8)	2 (3.8)	0 (0.0)	
9 am-5 pm	5 (9.6)	0 (0.0)	5 (9.6)	
Others	1 (1.9)	1 (1.9)	0 (0.0)	
Mean number of patients who received services:				
Dental filling	37.72 (37.7)	39.56 (40.7)	27.43 (8.66)	198.13 (0.00)
Extraction	132.8 (190.8)	148.41 (201.7)	39.14 (35.10)	0.195 (0.000)
Root canal treatment	28.00 (11.5)	30.52 (6.2)	16.14 (21.13)	12.12 (0.000)
Whether provider inform patients of procedure				6.555 (0.135)
Yes	51 (98.1)	45 (86.5)	6 (11.5)	
No	1 (1.9)	0 (0.0)	1 (1.9)	
Referral mechanism				3.279 (0.070)
Yes	37 (71.2)	30 (57.7)	7 (13.5)	
No	15 (28.8)	15 (28.8)	0 (0.0)	
*Where patients are usually referred to				2.663 (0.264)
Tertiary	29 (76.3)	22 (57.9)	7 (18.4)	
Secondary	1 (1.9)	1 (2.6)	0 (0.0)	
Private clinic	7 (21.1)	8 (21.1)	0 (0.0)	
Total	37 (100.0)	31 (81.6)	7 (18.4)	

Table 3 below shows that there is a difference in the availability of both disposable and reusable equipment across facility types (p < 0.05). The use of infection control guideline is similar across facility type (*p* < 0.05). A good number of respondents from both facility types indicated that they have functional equipment. All respondents from the private health facilities interviewed indicated the availability of clean water, soap and personal protective equipment compared to the public.

**Table 3:** Comparing available equipment and infection control measures in public and private dental facilities

Variables	Total f (%) N = 52	Public facility n (%)	Private facility n (%)	X^2^(P-value)
Equipment used in facilities				
Disposable	6 (11.5)	6 (11.5)	0 (0.0)	1.055 (0.580)
Reusable	2 (3.8)	2 (3.8)	0 (0.0)	0.324 (0.569)
Auto-disable	1 (1.9)	1 (1.9)	0 (0.0)	0.159 (1.000)
Both Disposable and Reusable	48 (92.3)	41 (78.8)	7 (13.5)	0.674 (1.000)
Use national infection control guideline				
Yes	42 (80.8)	36 (69.2)	6 (11.5)	7.853 (0.049)
No	1 (1.9)	1 (1.9)	1 (1.9)	
Can’t say	9 (17.3)	8 (15.4)	0 (0.0)	
Availability of functional equipment				
Electric autoclave	51 (98.1)	44 (84.6)	7 (13.5)	0.159 (1.000)
Electric heater sterilizer	38 (73.1)	31 (59.6)	7 (13.5)	2.980 (0.169)
Fun health sterilizer	36 (69.2)	29 (55.8)	7 (13.5)	3.595 (0.085)
Electric steamer	18 (32.7)	18 (34.6)	0 (0.0)	4.282 (0.039)
Pot with cover	14 (25.0)	12 (23.1)	2 (3.8)	0.011 (0.916)
Cold sterilization	34 (65.4)	28 (53.8)	6 (11.5)	1.477 (0.399)
X-ray machine	43 (82.7)	39 (75.0)	4 (7.7)	3.689 (0.055)
Dental syringe	52 (100.0	45 (86.5)	7 (13.5)	0.161 (1.021)
Light curing machine	46 (84.6)	39 (75.0)	7 (13.5)	1.055 (0.580)
Resources for infection control				
Clean water	50 (96.2)	43 (82.7)	7 (13.5)	0.324 (1.000)
PPE	51()98.1)	44 (84.6)	7 (13.5)	0.159 (0.690)
Soap	52 (100.0)	45 (86.5)	7 (13.5)	0.234 (0.873)

Table 4 below shows the cost of dental caries treatment procedure per patient. For a complete procedure, the average amount charged for consultation per patient is N1357 and N1580 in public and private facilities respectively. The average cost for the majority of procedures is much higher in private facilities.

**Table 4:** Cost of dental caries treatment procedures per patient

Variables	Public facilities Mean (SD)	Private facilities Mean (SD)	Levene’s test for equality of variances	t-test for equality of means (sig-2 tailed)	Total Mean (SD)
Registration	1357.1 (801.8)	1580.7 (648.5)	0.122	0.090	1550.0 (667.0)
Consultation	571.4 (534.5)	1113.6 (644.9)	0.770	0.007	1039.2 (653.8)
Drug	11.4 (75.4)	571.4 (534.5)	0.000	0.012	88.23 (277.6)
X-ray	976.7 (552.5)	1142.8 (556.3)	0.000	0.311	1000.0 (247.4)
Composite filling	6045.5 (1033)	9714.3 (955.9)	0.725	0.000	6549.0 (1616.3)
GIC filling	5090.9 (1654)	16,714.3 (1592.9)	0.580	0.000	6686.3 (7007.1)
Amalgam filling	4193.2 (947.6)	5333.3 (877.4)	0.000	0.000	4265.9 (966.0)
Extraction	4340.9 (491.5)	7857.1 (367.6)	0.657	0.000	4823.5 (1599.4)
Root canal	19,545.5 (3015)	31,214.3 (596.1)	0.432	0.000	21,147.1 (6221.2)
Porcelain Crown	34,932 (2002.4)	40,500.0 (2161.4)	0.154	0.513	35,600.0 (12,182.3)
Acrylic crown	11,714.3 (5964)	13,238.6 (4796.4)	0.350	0.077	13,029.4 (4116.3)
Bridge (Fixed)	11,486.1 (8392)	29,166.7 (14,288.7)	0.058	0.944	75,307.7 (74,713.2)
Partial denture	5261.4 (7171)	10,714.3 (11,455.9)	0.025	0.128	6009.8 (7972.8)
Scaling and polishing	1666.7 (1813)	6714.3 (3309.5)	0.828	0.023	3526.3 (3802.3)

The data shows that the T-test for consultation, composite and GIC fillings, extraction, root canal and scaling and polishing has a p-value of < 0.05, we, therefore, reject the null and accept the alternative hypothesis ***(H***_***1***_***)*** which states there is a significant difference in the cost of providing dental caries treatment service in the public and private dental facilities.

**Fig. 2:**
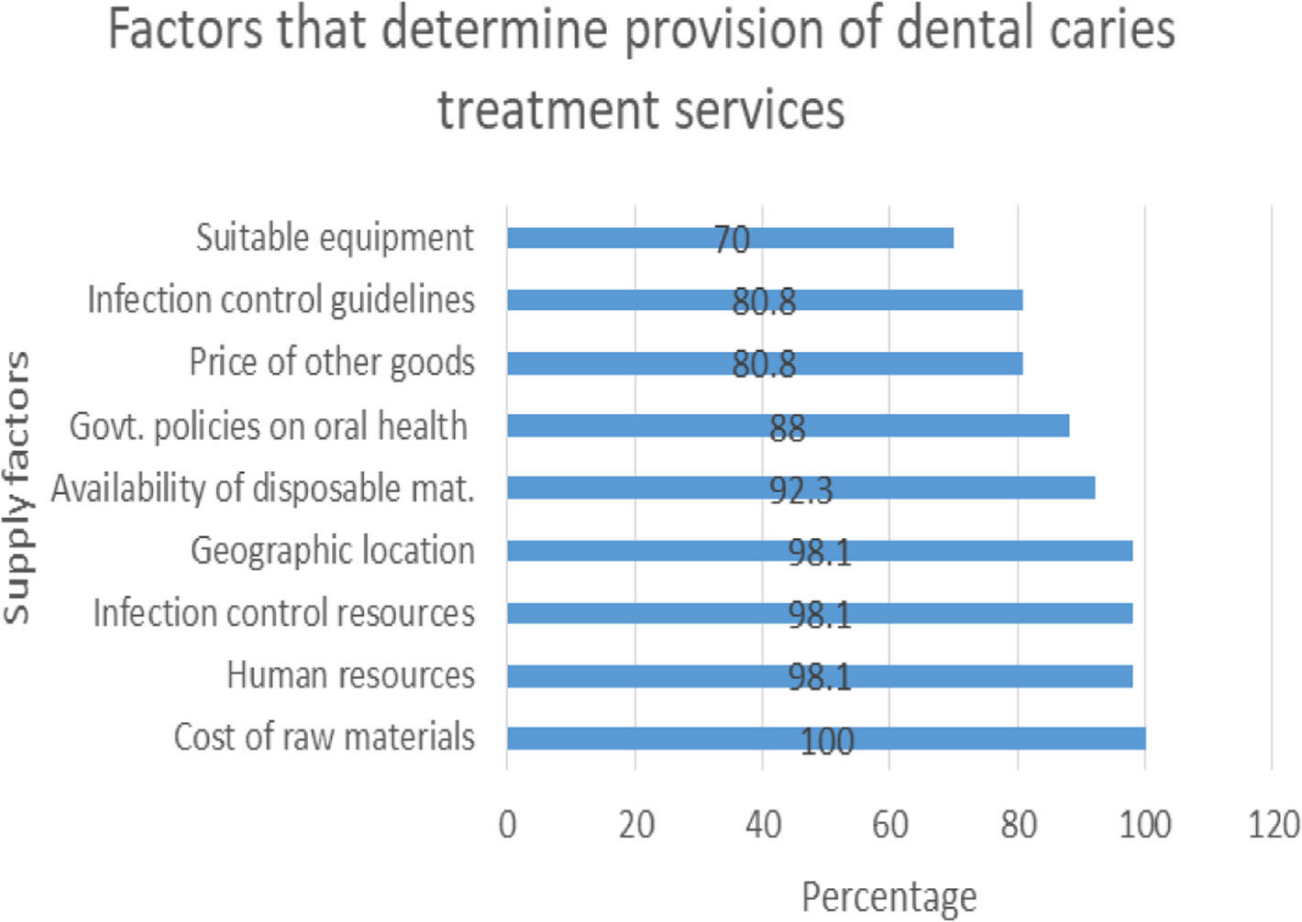
Factors that determine the provision of dental caries services

Figure 2 shows factors which influence the provision of dental caries treatment services in either public or private facilities: Cost of raw materials (100%) affects the cost of providing more and suitable equipment affects it the least (70%).

### Findings of qualitative interviews stated below show

#### Accessibility

##### Location of oral health services

Some respondents stated that facility location generally influences the type of services provided, the number of patients seen in a facility, and the type of patients seen. While some others, (mostly, respondents in the private dental facilities) opined that location will only influence the caliber of patients that attend a dental facility and not the type of services provided. However provision of a type of service is most times tailored to the ability to pay which is a reflection on the type of patient attending. Respondents also stated that the location of their facilities also determines the pricing of oral healthcare services. Below are the views of the study respondents:

*“We don’t get a large number of patients because the facility is far from town. When you add the transport cost with treatment cost and inconvenience of traveling to the teaching hospital, a lot of patients opt-out for clinics in town. (P6)*

*“Because of my location, respondents in the high socio-economic strata don’t like coming to my clinic. So that is when I refer them to other dentists with dental clinics situated in upper-class neighborhoods (P3).* A similar view was shared by another respondent however, in addition, the respondents say “*I am located in the village community and as such those that have some money will not want to visit a village dental center and often prefer the private dental clinics in town”(P1)*

*“Unfortunately we cannot have uniform pricing for dental caries treatment services among all dental facilities in both public and private. Even in private facilities prices sometimes differ because some of us cost services provided in our clinics based on whether we are situated in a high brow or low brow area” (P4).*

“*My price is partially based on location and is not about the price of the materials. We all buy dental materials from the same market so I feel the cost of my services is based more on location and my target clientele. My target market is not very rich because if they are rich I can adjust my prices at any time”.****(P2)***

#### Availability

##### Provision of services

In response to the question about the dental caries treatment mostly provided,the majority of the respondents stated that tooth extractions were the most common service provided in their facilities. Most of the respondents come so late with untreated dental caries that the only options left are root canals and tooth extractions. Because tooth extraction is the cheapest option, they usually opt for it. However, some dentists also claim that a good percentage of patients receive dental fillings. Root canal therapy, crowns, and dentures are treatment options for dental caries. Responses are shown below:

“*Extraction is the one that is regular. The patients may not be able to afford to pay for other ones, they will go for extraction. It is the cheapest”. (P1) We usually provide fillings and root canal therapy for bad cases but sometimes the patients cannot afford to pay despite our insistence so we might end up doing more extractions (P4)*

*“Most of them cannot pay because of their economic status. Most patients will always want to go for root canal but by the time you tell them how much root canal costs, they will end up telling you to extract (P3) “They usually say, I don’t have that kind of money” please remove the tooth.(P6)*

In looking at the factors influencing the provision of services, all the respondents state that the equipment and technological advances affect the type of treatment provided and also influences the pricing of services. The availability of electrical power source is a crucial issue in service provision because dental procedures need electricity it is difficult to provide services without adequate power supply. Majority of the respondents claim that the epileptic power supply has necessitated a rise in the cost of services to accommodate this. Only the public hospitals claim that their pricing remains the same even when there is no power.

“*… One of the major challenges we have is power supply (P3). Most times we have to provide a generator (P5). If there is no power, we use our generator and then continue treatment. But of course, that will now increase the cost of treatment because we now spend more providing power that the government should be giving us.”(P1)*

Most providers have an alternate power source which drives up cost. Respondents also claim that the type of equipment available in the facility will affect the ability to provide diverse treatment options for dental caries. Respondents in the rural public primary care facility are particularly affected by lack of equipment as most rural primary care facilities are grossly under-equipped. Respondent views are shown below:

*“Of course the type of equipment available affects the cost of services. Like fillings, we can’t do complex dental fillings. We do very simple fillings because of the type of equipment that we have affects it. We don’t have amalgamator for preparing our amalgams, we don’t have filling equipment so it affects. We should do simple filling here as primary rural health care. We don’t have them, we don’t have handpieces for cutting, and we don’t have bowls. So most times we just do GIC fillings or atraumatic restorative treatment which does not require us to use machines”(P7)*

Some other providers complained about the fluctuating or inordinate cost of dental materials affecting the cost of treatment. Dental service provision in most cases seems to be a monopoly in Enugu State and as such price-fixing by individual private dental practitioners is rampant. The dental facility heads set prices based on how cheap or expensive they purchased the consumable dental materials and what they perceive their profit margin should be.

*Cost of treatment is determined by the cost of materials but this is not immediate unless the management sees no returns” (P6). I usually charge based on the cost of the material. I can be lenient at times but if I see no returns I have to increase charges a bit so I can buy more materials. (P5)*

Government policies and taxes have also affected the cost of dental caries treatment services as well as every other dental service. Majority of the respondents state that since most of the equipment and consumables used in dental caries treatment are not manufactured locally, any increase in government tariff or importation ban, will drive the cost of equipment and products up and this will affect the pricing of services. However, this view was expressed mainly by the respondents in the private dental facilities as shown below:

“*Is just all these tariffs they place on importation. You know all the materials are almost imported. So it is difficult to get sometimes (P5). When the government increases tariff, the price of materials increases so you are bound to increase your own treatment cost. So we do not increase the price on our own but based on the cost of materials”.(P2)*

Let’s say it has affected negatively definitely because we have multiple taxations in Enugu State. Local government will tax you, the state government will tax you, and the environmental agency will tax you. All these things from one establishment and all these are still going back to government purse (P3). Multiple taxations are not encouraging. When you check how much you pay in a year plus the facility equipment and all those stuff. It is not easy and of course, the patient has to bear all these costs. (P4)

#### Affordability

##### Ability of consumers to pay

Patients that present at the dental clinic mostly pay out of pocket and only a few of the facilities offer health insurance for clients. Namely the tertiary institution, and a few private dental facilities in the urban area. The majority of dental health facilities do not cater to patients with health insurance. Dental treatment services under the National Health Insurance Scheme (NHIS) are listed as secondary care and as such dental health care providers are secondary care providers. Most dentists’ frown at this because it means the patients do not have immediate access to the dental health care provider and as such most patients turn up late. Other respondents opined that many dental health care providers and even patients have very poor knowledge of how the insurance scheme works for dentistry.

“*Well to be frank with us, NHIS has not really tried in the dental aspect. The most covered treatment choice is tooth extraction. If a patient opts out of an extraction, then any other treatment becomes too expensive. The cheapest any practitioner would want to do a root canal treatment is twenty thousand (20,000). NHIS doesn’t cover it and so that means they are encouraging patients to remove their teeth. So treatment option for anybody that is under NHIS is either scaling and polishing or extraction (P4). There are other insurance types, mainly private health insurance which is better but not available to everyone (P3).*

*‘We have NHIS, but to be honest, most patients still end up paying out of pocket, because the NHIS plan does not cover most of the treatment needed and even when they cover, the process is so tedious that some patients just opt to pay”(P6)*

“*: More awareness is needed and the insurance agencies should review their level of operation and then the population should stop being scared because most of them have this phobia for dental treatment and anything dental. No dentist can go to people’s houses to force them out. So I think information is key. Actually, the government should also review their own aspect and open more windows. (P5)*

Respondents opined also that the social health insurance scheme benefit package is very poor and policies regarding dental health insurance are basically non-existent or very poorly formulated as there is minimal awareness or knowledge about dental health insurance amongst HMOs and policymakers. This is reflected in the poor benefits package and the inability of most HMO’s to include dentistry in their health insurance plans. Only a few HMOs have relatively robust benefits packages and they are mainly private health insurance plans.

“*Well to be frank with you when it comes to dental policies most of my friends that are into health insurance and all whatnot that are doctors don’t really know anything about dental treatment and its policies. They are usually more interested in medical insurance. I keep telling them that the dental aspect is very important. They provide little or nothing and if you check very well those people that made the health financing policies are not even dentist” (P3).*

*“Yes. At the policy level, dentistry is important. In fact, at the policy level, it is very important because when you make things better, now so many people are going for the NHIS medically because they are seeing the benefit but most of them are not going in dental because they don’t see any benefit. Is either I wash my teeth or I remove it.” (P4)*

On the issue of fee exemptions and subsidies, most respondents are not aware of any government policy directive on this and usually only give discounts on compassionate grounds. Each facility grants indigent clients discounts based on their assessment of poverty status. There is no scientific way of deciphering this. Neither is there any laid down protocol for this. Waiver or subsidization of fees is dependent on the dentist and the management of the facility. Some practitioners, especially in the private sector, usually give the clients a payment plan where they can pay their fees in smaller regular installments. This is shown in the respondents’ statements below

*“Most times I do that out of empathy, not that there is a discount constitution. So my giving an exemption or price waiver depends on my interaction with the patient and if I can help” (P3).*

*“There is nothing like fee exemption or subsidy and I don’t think there is anything in the government policy that says that. If there is I have not seen” We just give a discount for some people period. Maybe the government hospital can give a full waiver but how can private do that? We are struggling too in this harsh economy (P4).*

*“In a public center, you do not have the power to give any waiver or subsidy unless it’s an already documented protocol in the center. If not permission must be sought and the go-ahead was given by management before that can be done” (P6)*

#### Accommodation

##### Organization of services

Excerpts from literature show that the right number of staff and appropriate staff mix goes a long way in improving service provision in healthcare facilities [16]. In response to the question of appropriate staffing, majority of the respondents claim to have an appropriate number of staff, with the right staff cadre to provide quality dental care. We observed that the public oral health facilities had a good complement of staff except for the primary care facility in the rural area. This had only one dentist catering to the population. The private facilities claimed to each have different staff cadres in their dental team making service provision better. However, on further probing, we observed that private clinics most times had one staff cadre performing multiple functions such as a dental therapist doubling as a dental surgery assistant. The human resource challenge in public dental facilities offering primary dental care in the rural areas is glaring, however this challenge also exists in the private facilities though muted. Quotes from the respondents are shown below.

*“Another thing I face is that you know they said two good heads are better than one so I’m all alone here. If I need a second opinion I don’t have anybody to turn to. So that is another challenge I’m facing. Besides the work is too much for one person. But because I am alone and greatly short-staffed. I don’t really take on too many patients and I don’t think the services provided here will ever meet the needs of the people unless the situation changes” (P7)*

*My staff strength is adequate I have two dental therapists and one double’s as a dental nurse and I have part-time technologists. (P4)*

*We have enough doctors and dental staff to meet the needs of our patients. We have the full complement. All staff cadres are ably represented. (P6)*

#### Awareness

##### Communication and information about oral health services

Most of the respondents believed that the majority of members of their community had little knowledge about dental caries treatment options and treatment of dental diseases as a whole. This they believe would account for late presentation of most patients with dental caries, which would have progressed so bad that the only option would be to do a root canal or extraction. As root canal is more expensive, a lot of them opt out for tooth extraction. Majority of the oral health facilities do not carry out dental awareness or oral health education programs with the exception of the teaching hospital through its community dentistry unit. Some of the private dental clinics claim though to carry out sporadic enlightenment programs in elementary schools in their environment. Most cite funding challenges to carry out mass oral health enlightenment programs.

*“Ok, the challenges I face are that most of the community members are not enlightened that is they are ignorant of dental care … (P7)*

*“We do conduct oral health education and awareness talks in some communities around us. Our community dentistry department sees to this angle (P6)*

*“We try to do oral health awareness but it’s mostly for patients that we have finished treating. It’s difficult to do community enlightenment because it’s cost-intensive, who will pay. Government hospitals can do that because the government will pay or subsidize, but we have to pay for everything from what we earn in the clinic-------- ah it’s difficult oh (P4)*

***“****Schools, we go to some local schools in our area and just talk to the children, but not all the time oh, we go maybe on children’s day or world oral health day. But no special arrangement just whenever we feel like”(P3)*

## Discussion

Findings from this study show there are a myriad of factors both contextual and individual that influence access to dental caries treatment services in Enugu State. These factors can either act as facilitators or barriers to the provision of dental caries treatment services. The low dentist to population ratio translates to inadequate human resources for oral health in the state and a maldistribution of available oral health care workers to favor urban locations more, translates to inadequate service provision in the rural areas and the state. The inadequate policy guidelines and funding of the Nigerian oral healthcare system translates to the often high cost of care as most providers’ especially private providers who finance and develop their clinics alone. Good oral health policies will translate to improved human resource development, infection control guidelines, and health financing structure for oral health.

There is a dearth of oral health facilities and a limited number of dentists in Enugu when compared with the number of persons in need of oral care. The large population and the number of dental clinics that exist are inadequate to meet the needs of the populace. Olusile et al. [7] posited that every local government should have a functional dental clinic to adequately meet the needs of the larger rural population [7]. Despite this recommendation, this anomaly in the distribution of oral health facilities in LGAs still exists. For instance, this study shows that within Nsukka environs (rural LGA in the study), there are about four oral health facilities (1 public and 3 private) and one dental laboratory. With a population of 417,700, this is inadequate and cannot meet the needs of the population. This is possibly the only rural primary oral health facility in a state with 17 LGAs. This is unlike general health facilities which abound in every local government area.

Geographical location was also found to determine the provision of services. This substantiates findings from a study that posited that oral health services are not readily accessible to the majority of the population in terms of geographical location [2]. However, some facilities located mostly in the rural areas, do not possess most of the equipment needed for dental treatment because of the rural nature of the area, the inadequate light source to operate machinery, and possibly non-availability of adequate security for dental equipment is a major challenge.

The availability of dental equipment and materials are quite necessary for the delivery of oral care [23]. The availability of equipment or lack of it can influence what services are provided by a facility. Lack of equipment can hamper the treatment options for patients. An oral health facility that only has equipment for extraction, will not be able to proffer options to the patient. This in a way, also will affect access/utilization. A study in eastern Africa [24] found that less than half of the facilities didn’t have the necessary materials consistently, and thus were unable to provide services required even if demanded by dental patients. A similar scenario was observed in this study, where the inability of one of the respondents to provide options for treating dental caries treatment was due to lack of equipment. The dentistwas unable to provide appropriate treatment and opted for an alternative that would suit the equipment available. One of the possible reasons for this unavailability could also be the cost of procurement and maintenance of requisite equipment. Thus poor equipping of oral health facilities in the state would ultimately deride the basis for quality and equitable oral health care which is one of the positive throwbacks of universal health coverage. Dental caries treatment services cannot be provided in a facility if the proper equipment and staff are unavailable.

The cost of services is a vital determinant to access [23, 25]. In our study, we observed that the majority of the dental caries treatment services provided were influenced by the demand of the consumers. This demand is usually tied to the cost of services. The cost of services is highest in private oral health facilities as against public facilities. One of the possible reason for this is that the private provider bears the whole cost of financing the dental clinic without any input from government and in a situation where the private facilities use an alternate power supply to augment or replace entirely power from the national grid, the cost of running dental treatment services with an alternate power source like a generator is usually incorporated into their service charge. In comparison, governments make large financial input into public dental facilities and so dental caries treatment services are provided to the public at subsidized rates, therefore making dental care cheaper in public facilities [2].

Equipment and materials required for the provision of oral health care are expensive to procure and the technical support for the maintenance of these, are not readily available or easily accessible. Cost of raw materials in the current study is one of the factors that determine the provision of any dental caries treatment service. The cost of raw materials could either delay the acquisition of such material therefore in effect hinder service provision or if purchased at a high cost, will affect service charge which could in effect reduce or hinder access to such services. In Nigeria where the majority of materials used for dental caries treatment services are imported, fluctuation in the tariff placed on importation or a hike in importation tax will drive up procurement cost and thus increases the cost for the services. The private facilities are the most affected by importation tax hike and fluctuations.

Some of the facilities used for this study have both social and private health insurance accreditations. However, we observed that the majority of these facilities do not operate any form of health insurance. The National Health Insurance Scheme (NHIS) which is a mandatory social health insurance scheme, is expected to provide additional funding for oral healthcare [13]. However because dental care is not under primary healthcare, dental clinics do not benefit from capitation. This non-funding of dental clinics by the scheme coupled with patient unreliability and HMO payment unreliability could also be a reason why the majority of the private dental clinics do not register as National health insurance providers. Another reason could be that the dental health benefits package under NHIS is so minimal that a lot of procedures that could be covered for dental caries treatment are not. All these will definitely affect the equitable provision of dental caries treatment services and inevitably reduce access for the low-income population groups. This observation is similar to that found in other studies [5, 20].

The number and skill mix of dental professionals as well as the physical presence of a dentist in the oral health facilities is very important to dental care delivery and as such facilities that do not have a requisite number of dental care professionals with a dentist available always, are unable to provide optimal service. In our study we observed that the public facility in the rural area has only one dentist who might not be available always, this will inadvertently disturb service provision. On the other hand, the private facilities also have only one dentist, however, most of them have a good complement of staff on paper, and as such patients can be attended to or referred appropriately. However, the observation minimal staff doing multiple functions is a mainstay. This will still compromise the quality of service but will enable practice owners to cut down expenditure.

The issue of skill mix also affects service provision if there are inadequately trained staff to manage dental equipment. For example, if the equipment is available and there are no staff or trained staff to operate it, the provision of service remains a problem. There is inadequate financial and human capacity to provide and manage oral health services. Some of the facilities in the current study are either not adequately staffed or lack proper skills mix of staff to provide the required services. A study presented a similar finding. Our study also found that the price and availability of dental materials, equipment, and skilled workforce are also important determinants for providing dental caries treatment services.

### Study limitations

This study focused on only provider information and as such no information from users of oral healthcare services was obtained.

## Conclusion

Access to oral health care is a major concern that affects all aspects of healthcare. Inadequate awareness, knowledge, cost, inadequately skilled staff, limited facilities, and treatment options are factors that determine dental service provision and equally influence access to oral health care. There is still a limited number of dental facilities in the country providing optimal and equitable oral health care services. In order to address this, the discourse of general health is incomplete without oral health.. Oral health facilities need to be properly equipped with materials, equipment, and appropriate skills mix of trained dental staff. There is also a need to encourage public-private partnership with regards to oral healthcare. This is to drive down excessive costs and reduce individual price setting by practice owners. A public-private partnership environment will also help regulate procedure prices and provide a means of offering financial aid to practices ensuring quality service.

As has been stated earlier, oral health is yet to be taken seriously in Nigeria and in Enugu State. In order to achieve universal coverage in oral health, oral health awareness needs to be taken seriously especially among policymakers so as to inform the development and proper equipping of oral health facilities in every LGA, deployment of adequate human resources and determination and implementation of a better health financing strategy for oral health. The development of a good oral health plan will also take into consideration public-private partnerships to aid the development of private oral health facilities and bolster oral health care services in the state.

## Data Availability

All datasets used or analyzed during the current study, are available from the corresponding author on reasonable request.

## Abbreviations

LGA: Local Government Area
